# Meta-analysis of neoadjuvant immunotherapy for non-metastatic colorectal cancer

**DOI:** 10.3389/fimmu.2023.1044353

**Published:** 2023-01-27

**Authors:** Long Zhou, Xiao-Quan Yang, Guang-yue Zhao, Feng-jian Wang, Xin Liu

**Affiliations:** ^1^ Department of Orthopedics, Shengjing Hospital of China Medical University, Shenyang, China; ^2^ Department of General Surgery, Liaoning Cancer Hospital & Institute, Cancer Hospital of China Medical University, Shenyang, Liaoning, China; ^3^ Department of Colorectal Surgery, Liaoning Cancer Hospital & Institute, Cancer Hospital of China Medical University, Shenyang, Liaoning, China

**Keywords:** neoadjuvant immunotherapy, non-metastatic colorectal cancer, meta-analysis, dMMR/MSI-H group, pMMR/MSS group

## Abstract

**Background:**

Immunotherapy has been approved for the treatment of metastatic colorectal cancer. The efficacy and safety of neoadjuvant immunotherapy for the treatment of non-metastatic colorectal cancer remains unclear. We tried to explore clinical effect of neoadjuvant immunotherapy in the treatment of non-metastatic colorectal cancer.

**Methods:**

We searched the databases (PubMed, Wanfang Embase, Cochrane Library and China National Knowledge Infrastructure databases) to obtain suitable articles up to September 2022. The primary outcomes of pathological complete response (pCRs), major pathological response (MPR), objective response rate (ORR), R0-resection and anus preserving rate were collected and evaluated. Secordary outcomes (pCRs and MPR) of subgroup analysis between deficient mismatch repair/microsatellite instability-high group (dMMR/MSI-H) and proficient mismatch repair/microsatellite stable group (pMMR/MSS) and outcomes for rectal cancer were analyzed for the final results.

**Results:**

We included ten articles and 410 cases of non-metastatic colorectal cancer with neoadjuvant immunotherapy. There were 113 (27.5%) cases with the dMMR/MSI-H status and 167 (40.7%) cases with the pMMR/MSS status. pCRs was found in 167/373 (44.6%) patients (ES: 0.49, 95% CI: 0.36 to 0.62, *P*<0.01, chi^2^ = 65.3, *P*<0.01, *I* ^2^ = 86.2%) and MPR was found in 194/304 (63.8%) patients (ES: 0.66, 95% CI: 0.54 to 0.78, *P*<0.01, chi^2^ = 42.55, *P*<0.01, *I* ^2^ = 81.2%) with the random-effects model and huge heterogeneity. In the subgroup analysis, pCRs was higher in the dMMR/MSI-H group than the pMMR/MSS group in the fixed-effects model with minimal heterogeneity (OR: 3.55, 95% CI: 1.74 to 7.27, *P*<0.01, chi^2^ = 1.86, *P*=0.6, *I* ^2^ = 0%). pCRs was found in 58/172 (33.9%) rectal cancer patients (ES: 0.33, 95% CI: 0.26 to 0.40, *P*<0.01, chi^2^ = 3.04, *P*=0.55, *I* ^2^ = 0%) with the fixed-effects model and little heterogeneity.

**Conclusion:**

Neoadjuvant immunotherapy could increase pCRs and MPR rate for non-metastatic colorectal cancer. Neoadjuvant immunotherapy could achieve better pCRs rate in dMMR/MSI-H group than in the pMMR/MSS group. Neoadjuvant immunotherapy could be another treatment option for non-metastatic colorectal cancer.

**Systematic review registration:**

https://www.crd.york.ac.uk/prospero/#myprospero, identifier CRD42022350523.

## Background

The incidence of colorectal cancer is high, and it brings a serious threat to human health (1). Neoadjuvant therapy has been widely used in the clinical treatment, and it is one of the important modes of colorectal cancer (2, 3). Neoadjuvant therapy for rectal cancer is currently based on neoadjuvant chemo(radio)therapy, while neoadjuvant therapy for colon cancer and resectable metastatic colorectal cancer is mostly based on chemotherapy drugs (4, 5). Neoadjuvant chemo(radio)therapy for rectal cancer is the classic mode of neoadjuvant therapy (6, 7). ORR (objective response rate) and pCR (pathological complete response) rate of colorectal cancer after neoadjuvant chemotherapy are 40% and 5% respectively, while the pCR rate of colorectal cancer after neoadjuvant chemo(radio)therapy is about 10%-15% (8, 9).

In 2015, the KEYNOTE-016 study (NCT01876511) indicated that dMMR/MSI-H metastatic colorectal cancer could significantly benefit from programmed death ligand-1 (PD-L1) monoclonal antibody immunotherapy (10). But for pMMR/MSS metastatic colorectal cancer, immunotherapy could not achieve similar clinical efficacy with dMMR/MSI-H metastatic colorectal cancer (11). Guidelines have recommended that immunotherapy is suitable for dMMR/MSI-H metastatic colorectal cancer, but there are no relevant guidelines recommending whether neoadjuvant immunotherapy could be used in non-metastatic colorectal cancer.

The original NICHE study cohort reported the final efficacy data at the 2022 ASCO Annual Meeting, it showed that 30% of pMMR/MSS patients and all the dMMR/MSI-H colorectal cancer patients could respond to neoadjuvant nivolumab plus ipilimumab (12). However, the clinical efficacy and scope of neoadjuvant immunotherapy for colorectal cancer remains unclear (13). Therefore, we collected relevant articles of neoadjuvant immunotherapy for non-metastatic colorectal cancer. We tried to explain the clinical effects of neoadjuvant immunotherapy for non-metastatic colorectal cancer and further compared the difference between dMMR/MSI-H group and pMMR/MSS group in the subgroup analysis.

## Methods

### Literature search

The protocol has been registered on the PROSPERO website (CRD42022350523, https://www.crd.york.ac.uk/prospero/#myprospero). The **Supplementary Material 1** showed the details. The meta-analysis was performed according to the PRISMA guidelines (**Supplementary Material 2**).

According to the design and purpose of the article, we conducted the relevant literature search in the Embase, PubMed, Cochrane Library, CNKI (China National Knowledge Infrastructure) and Wanfang databases (up to September 2022). The search terms were “colorectal cancer” and “neoadjuvant immunotherapy”.

The PICO model was followed to guide our literature research in the subgroup analysis: population, intervention, comparator and outcomes. The population included non-metastatic colorectal cancer patients. The intervention was dMMR/MSI-H group. The comparator was pMMR/MSS group. The outcomes included pCRs and MPR.

### Inclusion and exclusion criteria

The inclusion criteria were as follows: (1) non-metastatic colorectal cancer, (2) single-arm study, cohort study, prospective study, retrospective study and RCTs, (3) the included patients performed neoadjuvant immunotherapy.

The exclusion criteria were as follows: (1) metastatic colorectal cancer; (2) case reports, meeting, letter and other unsuitable types; (3) no neoadjuvant immunotherapy.

### Data extraction and quality assessment

Two reviewers (GYZ and FJW) searched the relevant literatures and sorted the useful clinical data independently with the help of the revised version of MINORS (methodological index for non-randomized studies) (14). The revised version of MINORS was used for the quality assessment of observational or non-randomized studies (15). The third reviewer (LZ) resolved the inconsistencies between the above two authors.

The relevant clinical data was shown by the tables. **Tables 1**–**4** showed the baseline data (such as sex, country, age, MMR status, tumor location and so on), the primary outcomes (MPR, pCRs, and so on), secondary outcomes (MPR and pCRs) and outcomes for rectal cancer (MPR, pCRs, and so on). The details of clinical stage and pathlologic stage were shown in the **Supplementary Materials 3**, **4** respectively. The details of postoperative complications, adverse events of neoadjuvant therapy and neoadjuvant immunotherapy plan were shown in **Supplementary Materials 5**–**7** respectively. We obtained no further information after we contacted the relevant authors of the included studies.

**Table 1:** Characteristics of the included articles.

Study	Country	Year	Case	Age	Sex (male/female)	ECOG (0-1)	Tumor diameter(cm)	Median distance from anal verge (cm)	CEA≥5 ng/mL
Bando H 2022	Japan	2022	44	60.6	29/15	44	NR	NR	NR
Chalabi M 2022	Netherlands	2020	40	61.9	18/22	40	NR	NR	NR
Hu H 2022	China	2022	34	49	23/11	34	NR	NR	NR
Kothari A 2022	America	2022	9	55.9	5/4	NR	NR	NR	NR
Li YJ 2021	China	2021	24	65	15/9	NR	5.1 (2.1-7.5)	4 (3-7)	NR
Lin Z 2021	China	2021	30	57	17/13	30	5.4 (2.1-10.0)	4.7 (1.9-9.0)	13
Liu ZX 2022	China	2022	94	58	48/46	94	NR	NR	NR
Rahma OE 2021	America	2021	90	NR	60/30	NR	NR	NR	NR
Shamseddine A 2020	Lebanon	2020	13	62.2	9/4	13	NR	10 (3-14)	NR
Zhang X 2022	China	2022	32	44	17/15	32	NR	NR	NR
Study	CRM	EMVI	MMR status			Tumor location			Lynch syndrome
			dMMR/MSI-H	pMMR/MSS	unknown	right colon	transverse colon	left colon	
Bando H 2022	NR	NR	5	39	0	0	0	44	NR
Chalabi M 2022	NR	NR	21	19	0	22	3	15	7
Hu H 2022	NR	NR	19	0	17	15	6	13	5
Kothari A 2022	NR	NR	9	0	0	6	0	3	3
Li YJ 2021	10	10	NR	NR	NR	0	0	24	NR
Lin Z 2021	NR	NR	1	28	1	0	0	30	NR
Liu ZX 2022	NR	NR	26	68	0	9	1	83	NR
Rahma OE 2021	NR	NR	NR	NR	NR	0	0	90	NR
Shamseddine A 2020	NR	NR	0	13	0	0	0	13	NR
Zhang X 2022	NR	NR	32	0	0	11	4	17	NR

dMMR, deficient mismatch repair; MSI-H, microsatellite instability-high; MSI-L, microsatellite instability-low; pMMR, proficient mismatch repair; MSS, microsatellite stable; CRM, circumferential resection margin; EMVI, extramural venous invasion; pCR, pathological complete response; MPR, major pathological response; ORR, objective response rate; cCR, complete clinical response; NR, no record.

The orders of additional information were range, standard deviation, percentage or NR (if not reported).

**Table 2:** primary outcomes.

Study	MPR (%)	pCRs (%)	R0 resection (%)	anus preserving rate (%)	ORR (%)	cCR (%)
Bando H 2022	17 (38.8)	14 (31.8)	NR	NR	NR	NR
Chalabi M 2022	23 (65.7)	14 (40)	NR	NR	NR	NR
Hu H 2022	29 (85.5)	26 (76.4)	34 (100)	NR	NR	NR
Kothari A 2022	9 (100)	8 (88.8)	NR	NR	5 (55.5)	NR
Li YJ 2021	10 (50)	6 (30)	19 (95)	16 (80)	18 (75)	3 (12.5)
Lin Z 2021	18 (66.6)	13 (48.1)	27 (100)	24 (99.0)	NR	NR
Liu ZX 2022	57 (60.6)	39 (41.5)	94 (100)	NR	83 (88.3)	NR
Rahma OE 2021	NR	22 (31.9)	65 (94)	41 (59.4)	NR	11 (13.9)
Shamseddine A 2020	6 (50)	3 (25)	NR	NR	NR	NR
Zhang X 2022	25 (86.2)	22 (75.9)	29 (100)	NR	29 (100)	3 (9.4)
Total	194 (63.8)	167 (44.6)	268 (98.5)	81 (69.8)	135 (88.9)	17 (14.1)

dMMR, deficient mismatch repair; MSI-H, microsatellite instability-high; MSI-L, microsatellite instability-low; pMMR, proficient mismatch repair; MSS, microsatellite stable; CRM, circumferential resection margin;

EMVI, extramural venous invasion; pCR, pathological complete response; MPR, major pathological response;

ORR, objective response rate; cCR, complete clinical response; NR, no record.

The orders of additional information were range, standard deviation, percentage or NR (if not reported).

**Table 3:** secondary outcomes.

Study	MPR (%)		pCRs (%)	
	dMMR/MSI-H	pMMR/MSS	dMMR/MSI-H	pMMR/MSS
Bando H 2022	3 (60)	14 (37.8)	3 (60)	11 (29.7)
Chalabi M 2022	19 (95)	4 (27)	12 (60)	2 (13.5)
Hu H 2022	NR	NR	NR	NR
Kothari A 2022	9 (100)	0	8 (88.9)	0
Li YJ 2021	NR	NR	NR	NR
Lin Z 2021	1 (100)	17 (65.4)	1 (100)	12 (46.2)
Liu ZX 2022	17 (65.4)	40 (58.8)	15 (57.7)	24 (35.3)
Rahma OE 2021	NR	NR	NR	NR
Shamseddine A 2020	NR	6 (50)	NR	3 (25)
Zhang X 2022	25 (86.2)	0	22 (75.9)	0

dMMR, deficient mismatch repair; MSI-H, microsatellite instability-high; MSI-L, microsatellite instability-low; pMMR, proficient mismatch repair; MSS, microsatellite stable; CRM, circumferential resection margin; EMVI, extramural venous invasion; pCR, pathological complete response; MPR, major pathological response;

ORR, objective response rate; cCR, complete clinical response; NR, no record.

The orders of additional information were range, standard deviation, percentage or NR (if not reported).

**Table 4:** outcomes for rectal cancer.

Study	MPR (%)	pCRs (%)	R0 resection (%)	anus preserving rate (%)	ORR (%)	cCR (%)
Bando H 2022	17 (38.8)	14 (31.8)	NR	NR	NR	NR
Li YJ 2021	10 (50)	6 (30)	19 (95)	16 (80)	18 (75)	3 (12.5)
Lin Z 2021	18 (66.6)	13 (48.1)	27 (100)	24 (99.0)	NR	NR
Rahma OE 2021	NR	22 (31.9)	65 (94)	41 (59.4)	NR	11 (13.9)
Shamseddine A 2020	6 (50)	3 (25)	NR	NR	NR	NR
Total	51 (49.5)	58 (33.9)	111 (96.5)	81 (69.8)	18 (75)	14 (13.6)

dMMR, deficient mismatch repair; MSI-H, microsatellite instability-high; MSI-L, microsatellite instability-low; pMMR, proficient mismatch repair; MSS, microsatellite stable; CRM, circumferential resection margin; EMVI, extramural venous invasion; pCR, pathological complete response; MPR, major pathological response;

ORR, objective response rate; cCR, complete clinical response; NR, no record.

The orders of additional information were range, standard deviation, percentage or NR (if not reported).

### Statistical analysis

Stata 11.0 and RevMan 5.0 software was used to analyze the dichotomous data, and it was evaluated by relative risks (ORs or RRs) with 95% confidence intervals. Random effects models and fixed effects model were used to analyse the data with huge heterogeneity (*I* ^2^≧50%) and for little heterogeneity (*I* ^2^<50%) respectively. Publication bias was assessed by the funnel plots.

## Results

### Study selection

**Figure 1:**
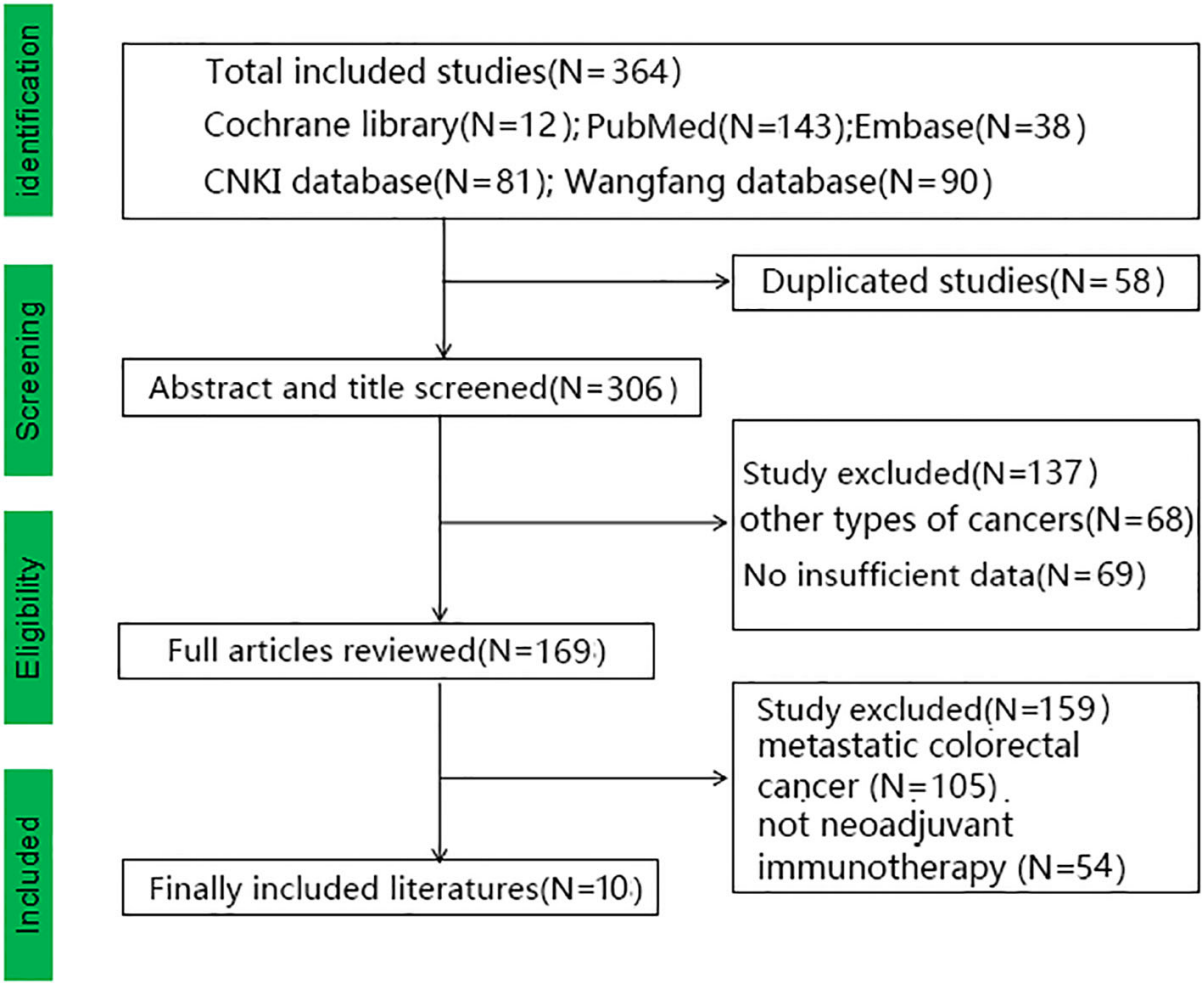
Study selection followed by PRISMA diagram.

364 relevant studies were obtained after medical database searching. After we remove the duplicate literatures (N=58), not non-metastatic colorectal cancer (N=105), not neoadjuvant immunotherapy (N=54) and other literatures (**Figure 1**). We finally included ten articles with 410 non-metastatic colorectal cancers. There were 113 (27.5%) cases with the dMMR status and 167 (40.7%) cases with the pMMR status, while 130 (31.7%) cases remain unknown MMR status (16–25). In the subgroup analysis, the patients of Rahma OE 2021 and Li 2021 do not know the MMR status, Kothari A 2022, Shamseddine A 2020 and Zhang X 2022 are all belong to the dMMR status, so the above studies cannot be included in the subgroup analysis. **Table 1** and **Supplementary Materials 3**, **4** showed the baseline data of the included studies. Eight English study and two Chinese studies were included, all the included studies achieved 12 points with high-moderate quality according to MINORS standard, the literature quality scores and the specific informations were in the **Supplementary Material 8**.

The vast majority of neoadjuvant therapy regimens are neoadjuvant chemoradiotherapy (long-course radiotherapy: 4500 cGy in 25 fractions or short-course radiotherapy: 2500 cGy in 25 fractions with or without CAPOX and FOLFOX) plus pembrolizumab, ipilimumab or nivolumab (200 mg) for local advanced rectal cancer. Avelumab (10 mg/kg), toripalimab (3 mg/kg) and camrelizumab (200 mg) were immunotherapy drugs in Shamseddine A 2020, Hu H 2022 and Lin Z 2021 respectively. The specific informations were in the **Supplementary Material 7**. Pembrolizumab, ipilimumab or nivolumab (200 mg) are mainly immunotherapy drugs for local advanced rectal cancer. Pembrolizumab and ipilimumab are mainly immunotherapy drugs for colon cancer. The proportion of clinical stage III patients before neoadjuvant immunotherapy was 77.2%, and the proportion of clinical stage III patients after neoadjuvant immunotherapy decreased to 20.7%. The proportion of T3-T4 patients and N1-N2 patients before neoadjuvant immunotherapy was 98.2% and 81.8% respectively, while the proportion of T3-T4 patients and N1-N2 patients after neoadjuvant immunotherapy was 18.3% and 12.6% respectively (**Supplementary Materials 3**, **4**). According to the RECIST criteria, ORR was 88.9% after neoadjuvant immunotherapy. pCRs and cCR was observed in 167(44.6%) and 17(14.1%) patients after neoadjuvant immunotherapy(**Table 2**). **Table 2** showed the information of primary outcomes (pCRs, MPR, ORR, R0 resection and anus preserving rate). **Table 3** showed the information of secondary outcomes (pCRs and MPR) between dMMR/MSI-H group and pMMR/MSS group. **Table 4** showed the information of outcomes for rectal cancer.

### Primary outcomes: pCRs, MPR, ORR, R0-resection and anus preserving rate

**Figure 2:**
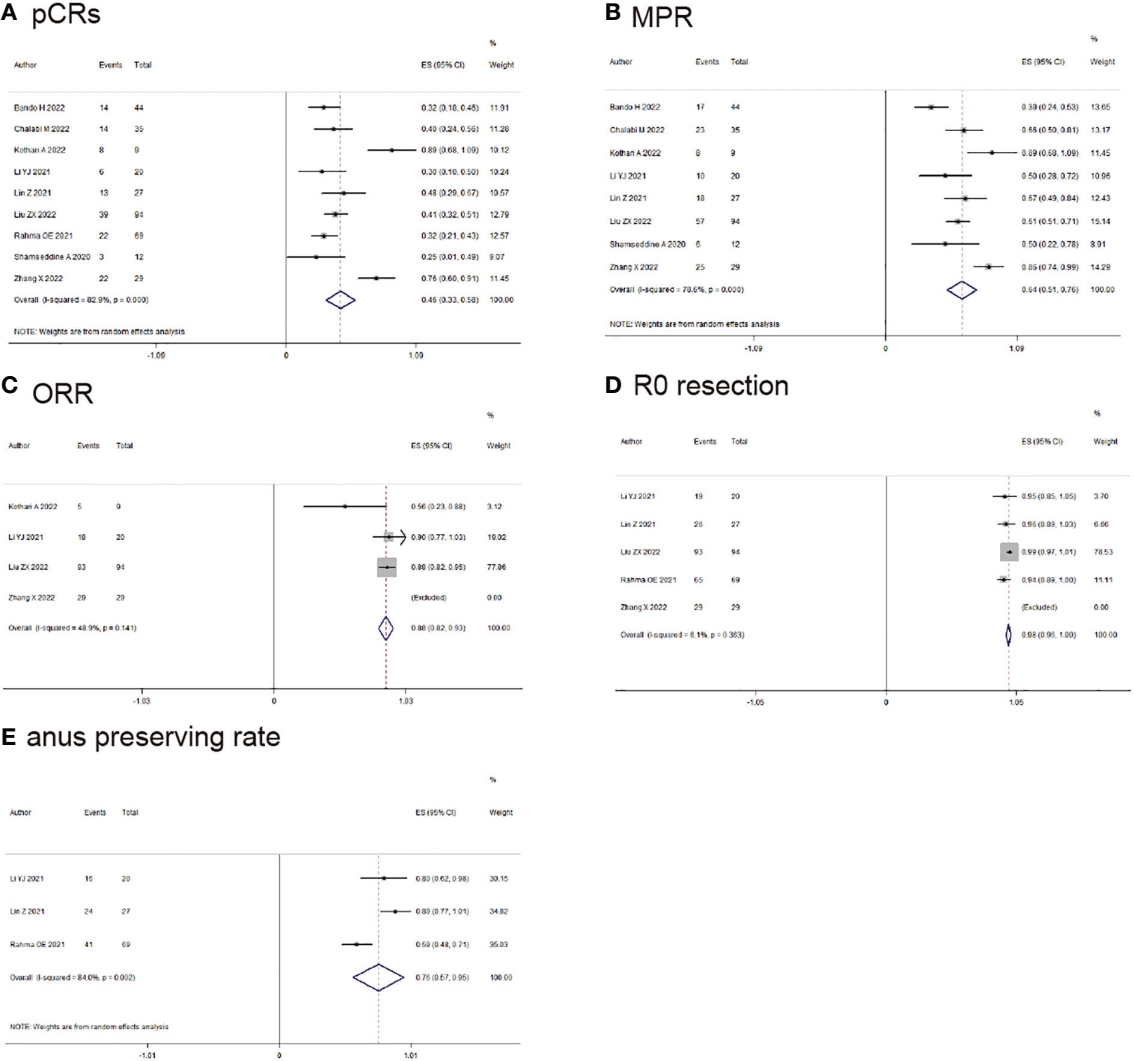
Primary outcomes of neoadjuvant immunotherapy for non-metastatic colorectal cancer. **(A)** pathological complete response (pCRs); **(B)** major pathological response(MPR); **(C)** objective response rate (ORR); **(D)** RO-resection; **(E)** anus preserving rate.

10 studies reported the clinical data of pCRs, pCRs was found in 167/373 (44.6%) patients (ES: 0.49, 95% CI: 0.36 to 0.62, *P*<0.01, chi^2^ = 65.3, *P*<0.01, *I* ^2^ = 86.2%, **Figure 2A**) with the random-effects model and huge heterogeneity. MPR was reported by 9 studies, MPR was found in 194/304 (63.8%) patients (ES: 0.66, 95% CI: 0.54 to 0.78, *P*<0.01, chi^2^ = 42.55, *P*<0.01, *I* ^2^ = 81.2%, **Figure 2B**) with the random-effects model and little heterogeneity.

ORR was reported by 4 studies, MPR was found in 135/152 (88.9%) patients (ES: 0.88, 95% CI: 0.82 to 0.93, *P*<0.01, chi^2^ = 3.92, *P*=0.14, *I* ^2^ = 48.9%, **Figure 2C**) with the fixed-effects model and little heterogeneity. R0-resection rate was found in 6 studies, R0-resection was found in 268/272 (98.5%) patients (ES: 0.98, 95% CI: 0.96 to 0.99, *P*<0.01, chi^2^ = 3.48, *P*=0.36, *I* ^2^ = 0%, **Figure 2D**) with the fixed-effects model and little heterogeneity. Anus preserving rate was reported by 3 studies, anus preserving rate was found in 81/116 (69.8%) patients (ES: 0.76, 95% CI: 0.57 to 0.95, *P*<0.01, chi^2 =^ 12.53, *P*<0.01, *I* ^2^ = 84%, **Figure 2E**) with the random-effects model and huge heterogeneity.

### Secondary outcomes (subgroup analysis): pCRs and MPR (dMMR/MSI-H vs pMMR/MSS group)

**Figure 3:**
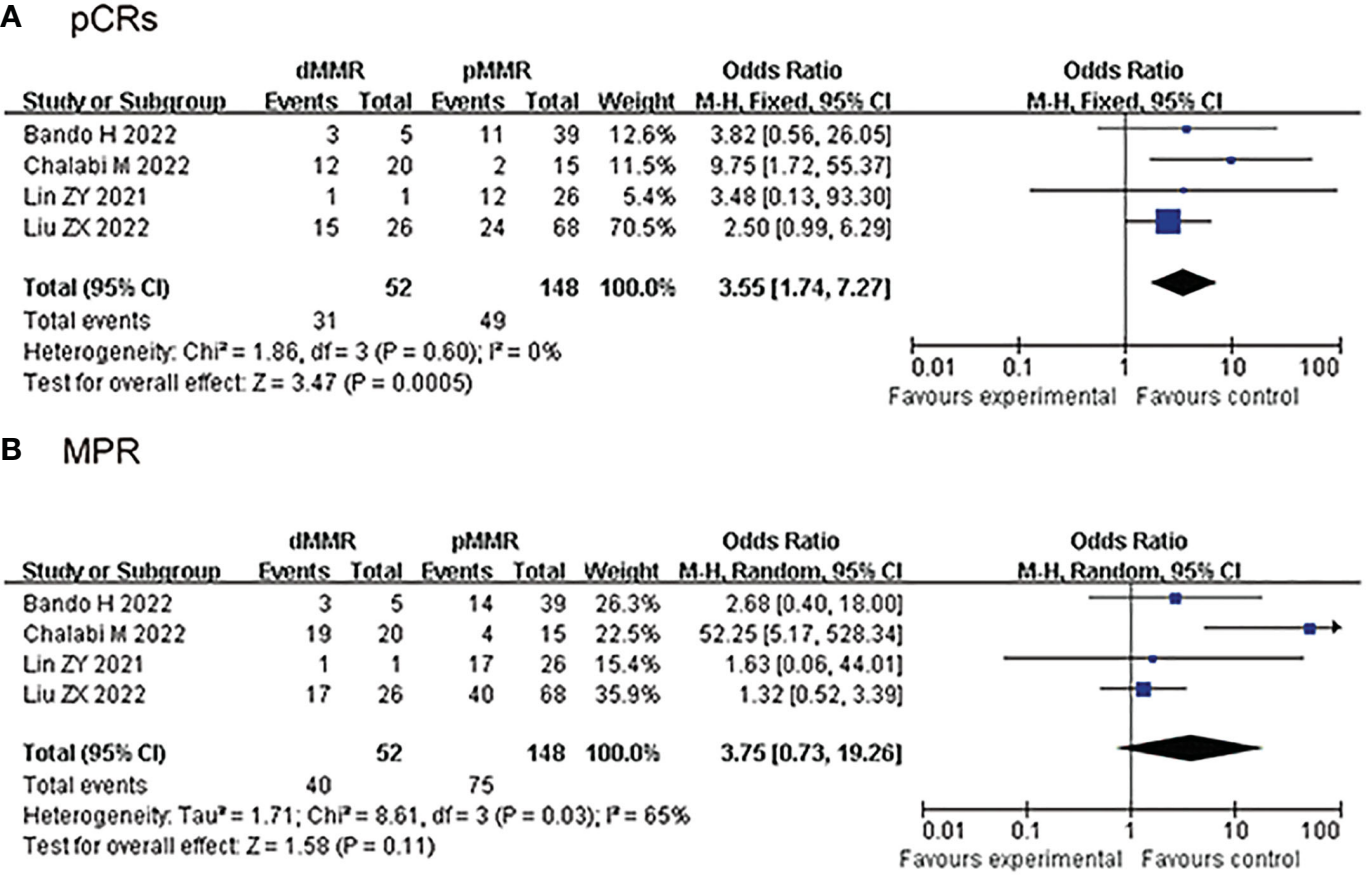
Secondary outcomes of neoadjuvant immunotherapy for non-metastatic colorectal cancer. (dMMR/MSl-H vs pMMR/MSS group). **(A)** pathological complete response (pCRs); **(B)** major pathological response (MPR).

4 studies reported the clinical data of pCRs for dMMR/MSI-H and pMMR/MSS group, pCRs was higher in the dMMR/MSI-H group than the pMMR/MSS group in the fixed-effects model with minimal heterogeneity (OR: 3.55, 95% CI: 1.74 to 7.27, *P*<0.01, chi^2^ = 1.86, *P*=0.6, *I* ^2^ = 0%, **Figure 3A**). 4 studies reported the clinical data of MPR for dMMR/MSI-H and pMMR/MSS group, MPR was similar in the dMMR/MSI-H group and the pMMR/MSS group in the random-effects model with huge heterogeneity (OR: 3.75, 95% CI: 0.73 to 19.26, *P*=0.11, chi^2^ = 8.61, *P*=0.03, *I* ^2^ = 65%, **Figure 3B**).

### Outcome for rectal cancer (pCRs, MPR, R0-resection and Anus preserving rate)

**Figure 4:**
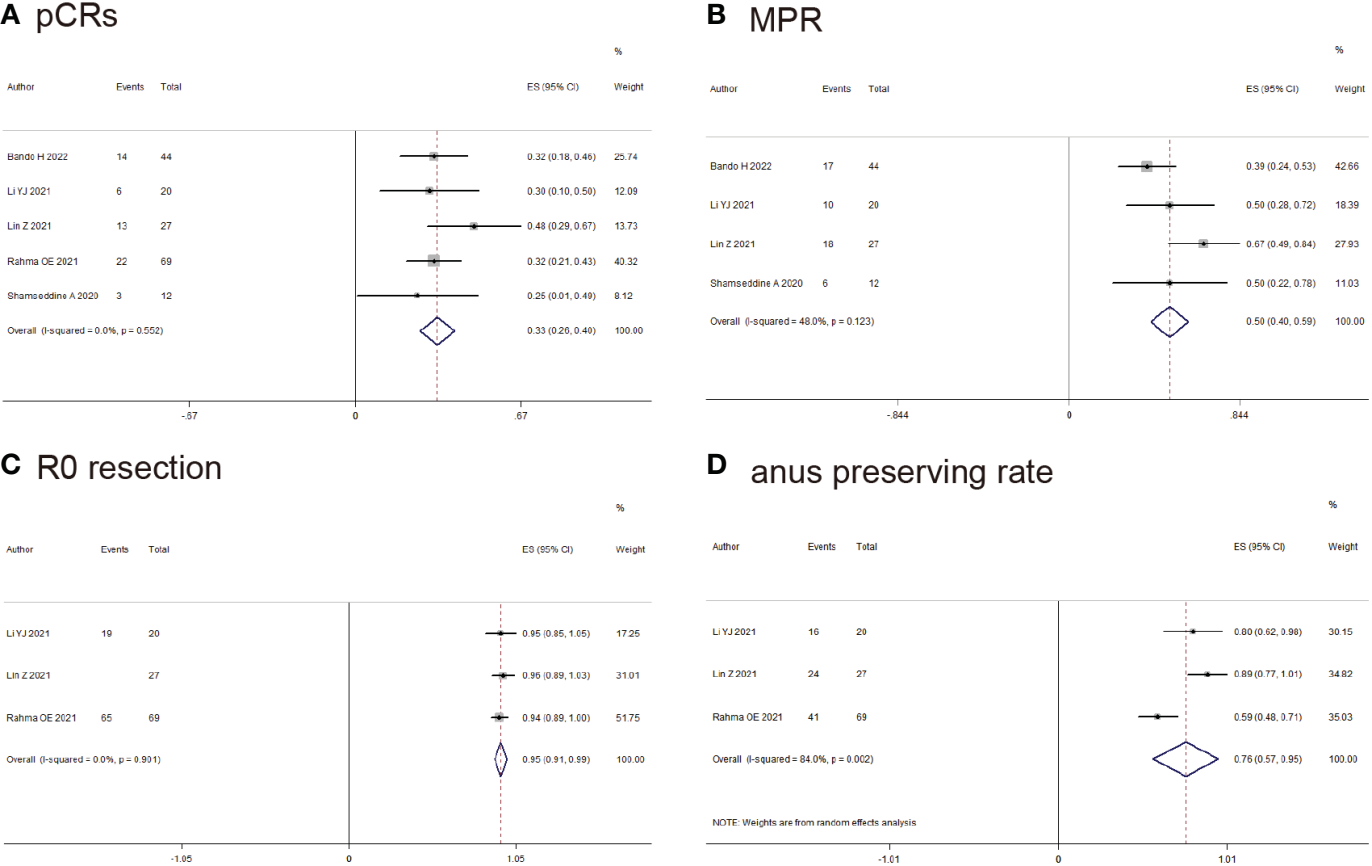
Outcomes of neoadjuvant immunotherapy for rectal cancer. **(A)** pathological complete response (pCRs); **(B)** major pathological response (MPR); **(C)** RO-resection; **(D)** Anus preserving rate.

5 studies reported pCRs of rectal cancer, pCRs was found in 58/172 (33.9%) patients (ES: 0.33, 95% CI: 0.26 to 0.40, *P*<0.01, chi^2^ = 3.04, *P*=0.55, *I* ^2^ = 0%, **Figure 4A**) with the fixed-effects model and little heterogeneity. 4 studies reported MPR of rectal cancer, MPR was found in 51/103 (49.5%) patients (ES: 0.50, 95% CI: 0.40 to 0.59, *P*<0.01, chi^2^ = 5.77, *P*=0.12, *I* ^2^ = 48%, **Figure 4B**) with the fixed-effects model and little heterogeneity. R0-resection was found in 111/116 (96.5%) patients (ES: 0.95, 95% CI: 0.91 to 0.99, *P*<0.01, chi^2^ = 0.21, *P*=0.90, *I* ^2^ = 0%, **Figure 4C**) with the random-effects model and huge heterogeneity. Anus preserving rate was found in 81/116 (69.8%) patients (ES: 0.76, 95% CI: 0.57 to 0.95, *P*<0.01, chi2 = 12.53, *P*<0.01, *I* ^2^ = 84%, **Figure 4D**) with the random-effects model and huge heterogeneity.

### Publication bias

The publication bias was visualized by RevMan 5.0 software with the clinical date of pCRs rate. We found that the points were evenly distributed in the forest plot.

## Discussion

Tumor immunity refers to a series of normal physiological processes in which the immune system in the body recognizes and kills tumor cells (26). Tumor cells could express checkpoint inhibitors (PD-L1 molecules) by themselves. When PD-L1 binds to PD-1, it decreased the activation of T cells around the tumor and immune attack of cells in the body, and finally induced the tumor immune escape (27). However, PD-L1 or PD-1 monoclonal antibody could block tumor immune escape and restore the anticancer function of the autoimmune system (28). Tumor immunotherapy has been widely used in clinical treatment, and has achieved good clinical effects in many kinds of cancers (29). KEYNOTE-177 study also confirmed that immunotherapy could improve ORR and survival time in dMMR/MSI-H metastatic colorectal cancer. Some guidelines have pointed out that immunotherapy could be used for metastatic colorectal cancer, especially for dMMR/MSI-H metastatic colorectal cancer (30).

But there are few studies focusing on neoadjuvant immunotherapy for non-metastatic colorectal cancer, and the value of neoadjuvant immunotherapy for non-metastatic colorectal cancer is not fully elucidated (13, 31). We tried to use the available clinical data and explain the clinical effects of neoadjuvant immunotherapy for non-metastatic colorectal cancer.

## Novelty of the study

Firstly, the study attempted to evaluate the effect of neoadjuvant immunotherapy for non-metastatic colorectal cancer. There was no similar meta-analysis, only several reviews presented the overview of neoadjuvant immunotherapy for non-metastatic colorectal cancer. Second, we compared the effects of neoadjuvant immunotherapy in the dMMR/MSI-H group and pMMR/MSS group in the subgroup analysis. Third, the meta-analysis explored many other outcomes (pCRs, MPR, ORR, R0-resection and Anus preserving rate) to clarify the advantages and disadvantages of neoadjuvant immunotherapy for non-metastatic colorectal cancer.

## Outcome results

### pCRs, MPR, ORR, R0-resection and Anus preserving rate

The pCR rate of neoadjuvant chemotherapy for colorectal cancer was about 5%, and the pCR rate of neoadjuvant chemoradiotherapy for rectal cancer was about 10-15% (5, 32). In our study, pCR rate of neoadjuvant immunotherapy was 38.3% for non-metastatic colorectal cancer patients. The ORR rate of neoadjuvant chemotherapy for colorectal cancer was about 40%, while ORR of neoadjuvant immunotherapy was 86.1% in our study. MPR of neoadjuvant immunotherapy was 58% in our study. Compared with the clinical statistics of neoadjuvant chemotherapy and neoadjuvant chemoradiotherapy, ORR and pCR rate of neoadjuvant immunotherapy were significantly improved. In the Rahma OE study, it was further confirmed that neoadjuvant immunotherapy had higher pCR rate and R0 resection rate than neoadjuvant CRT group. Although many meetings and case reports also had affirmed the clinical effect of neoadjuvant immunotherapy, there were little clinical controlled studies for the comparison between neoadjuvant chemoradiotherapy and neoadjuvant immunotherapy (33, 34). Due to the limited data we collected, there may be some bias in the final results. There are many ongoing clinical trials (clinical controlled studies about neoadjuvant chemoradiotherapy and neoadjuvant immunotherapy), we expected to the announcement of the final results, which can provide more recommendations for clinical treatment. R0-resection rate and anus preserving rate were 98.5% and 69.8% respectively, while cCR rate was 14.1%. The results of study indicated that neoadjuvant immunotherapy could improve tumor regression and pathological remission.

### dMMR/MSI-H versus pMMR/MSS (pCRs and MPR)

dMMR/MSI-H status was a unique, biomarker-selected type of colorectal cancer and it accounted for approximately 12% to 15% of all colorectal cancer patients. It is more prevalent in the right colon with poorly differentiated or mucinous adenocarcinoma, while only 2% of rectal cancer patients have dMMR/MSI-H status (35). Al-Sukhni et al. reported that the pCR rate of rectal cancer in the pMMR/MSS group and dMMR/MSI-H group were 8.9% and 5.9% after neoadjuvant chemoradiotherapy respectively (36). In our study, pCR and MPR rate in the dMMR/MSI-H group were 63.9% and 80.3% after neoadjuvant immunotherapy, respectively. While pCR and MPR rate in the pMMR/MSS group were 32.7% and 51.2% after neoadjuvant immunotherapy, respectively. Some studies reported that dMMR/MSI-H colorectal cancer patients were mostly insensitive to neoadjuvant chemo(radio)therapy (37). dMMR/MSI-H colorectal cancer has higher TMB (tumor mutation burden), and there are a large number of immune cells in tumor tissue, which is more suitable for immunotherapy (38). Based on above results, we speculated that neoadjuvant immunotherapy could be applied to non-metastatic colorectal cancer patients, especially for dMMR/MSI-H non-metastatic colorectal cancer, and it could improve pCR and MPR rates in the non-metastatic colorectal cancer patients.

### dMMR/MSI-H versus pMMR/MSS (immunotherapy alone vs immunotherapy+nC(R)T)

Three articles reported the clinical data of neoadjuvant immunotherapy alone, while six articles reported the clinical data of neoadjuvant immunotherapy combined with neoadjuvant chemo(radio)therapy (**Supplementary Material 9**). The MPR and pCR rates with the dMMR/MSI-H status were 81.3% and 65.3% in the neoadjuvant immunotherapy alone group, respectively. While the MPR and pCR rate with the pMMR/MSS status were 27% and 13.5% in the neoadjuvant immunotherapy alone group, respectively. The MPR and pCR rates with the dMMR/MSI-H were 86.6% and 80% in the neoadjuvant immunotherapy combined with neoadjuvant chemo(radio)therapy group, respectively. While the MPR and pCR rates with the pMMR/MSS status were 68.1% and 34.9% in the neoadjuvant immunotherapy combined with neoadjuvant chemo(radio)therapy group, respectively. Based on the above results, we speculated that non-metastatic colorectal cancer with the dMMR/MSI-H status is more likely to benefit from immunotherapy, neoadjuvant immunotherapy combined with neoadjuvant chemo(radio)therapy could achieve better clinical results than neoadjuvant immunotherapy alone. Several ongoing clinical trials are immunotherapy combined with neoadjuvant chemo(radio)therapy. We expected that the results of the ongoing clinical researches could find out the direction of immunotherapy treatment mode and suitable population (39).

### Neoadjuvant immunotherapy for rectal and colon cancer

Among the included articles for the neoadjuvant immunotherapy of colorectal cancer, there were several related studies about rectal cancer. We used the available data to perform statistical analysis. The rate of pCRs and MPR were 33.9% and 49.5% respectively, while R0-resection and anus preserving rate were 96.5% and 69.8% respectively (**Table 4**). In our study, the pCRs rate of neoadjuvant immunotherapy (33.9%) for rectal cancer was higher than the pCR rate (5-15%) of neoadjuvant chemo(radio)therapy. R0-resection, anus preserving rate and other index of neoadjuvant immunotherapy were similar with neoadjuvant chemo(radio)therapy for rectal cancer. Therefore, on the premise of timely controlling the adverse events of neoadjuvant immunotherapy, we speculated that neoadjuvant immunotherapy could improve the pCR rate and pathological response.

However, there are little related studies about colon cancer among the included articles. We can not collect available data for statistical analysis. NICHE study focused on early-stage colon cancer, the rate of pCRs and MPR were 40% and 65.7% respectively. In our study, the pCRs rate of neoadjuvant immunotherapy (40%) for colon cancer was also higher than the pCR rate (5-15%) of neoadjuvant chemo(radio)therapy. However, due to little literatures of colon cancer for neoadjuvant immunotherapy, the results of NICHE study could provide some reference for clinical work. More studies are needed to clarify the clinical effect of neoadjuvant immunotherapy for non-metastatic colorectal cancer.

### Neoadjuvant immunotherapy plan and tumor response to neoadjuvant immunotherapy

In the included literatures, the neoadjuvant immunotherapy regimens were mostly single PD-1 monoclonal antibody (ipilimumab, nivolumab or other PD-1 monoclonal antibody, 200mg, 2-6 cycles), while the regimen of the NICHE study was the combination of PD-1 monoclonal antibody (single ipilimumab 1 mg/kg and two nivolumab 3 mg/kg treatments). Neoadjuvant immunotherapy combined with neoadjuvant radiotherapy (SCRT or IMRT, 25-50.4 Gy) was widely used in rectal cancer, while neoadjuvant immunotherapy combined with neoadjuvant chemotherapy (FOLFOX or CAPOX) was widely used in colon cancer (**Supplementary Material 7**) Neoadjuvant immunotherapy could achieve effective clinical treatment effect for the dMMR/MSI-H non-metastatic colorectal cancer. Neoadjuvant immunotherapy combined with chemo(radio)therapy could be more helpful for for pMMR/MSS non-metastatic colorectal cancer. Based on the above results, the pCR rate, MPR rate, the proportion of III patients, T3-T4 patients and N1-N2 patients were significantly improved after neoadjuvant immunotherapy.

In Liu ZX 2022 et al, 26 patients with dMMR/MSI-H status were treated with neoadjuvant immunotherapy, the pCR rate was 57.7% (15/26) and the MPR rate was 65.4% (17/26). Among the 68 patients with pMMR/MSS status who received immunotherapy combined with neoadjuvant chemo(radio)therapy, the pCR rate was 35.3% (24/68), and the MPR rate was 58.8% (40/68). At present, whether neoadjuvant immunotherapy was suitable for neoadjuvant treatment of colorectal cancer, which neoadjuvant immunotherapy regimens was more suitable for colorectal cancer, whether neoadjuvant immunotherapy requires combined chemo(radio)therapy was still inconclusive, and further research was needed to explore.

### Adverse events and postoperative complications

The most common immune-related adverse events of immune checkpoint inhibitors (ICPIs) were skin disease (44%-68%), followed by gastrointestinal reactions (5%-50%), abnormal liver function (incidence) and endocrine disorders (6%) (40). The included studies also made the similar conclusions, while most immune-related adverse events were mild events (I-II grade). The occurrence of adverse events could be related to the overactivation of T lymphocytes. Mild adverse events can be treated symptomatically, while severe adverse events require discontinuation of immunotherapy time, hormone replacement therapy and other treatment options (41). Therefore, the options of the suitable immunotherapy drug, dosage and administration time can effectively avoid the occurrence of adverse events. The adverse reactions of neoadjuvant chemo(radio)therapy are mainly leukopenia, elevated transaminases, gastrointertinal disorders. The adverse reactions of neoadjuvant chemo(radio)therapy are mostly mild reactions, and many patients could successfully complete neoadjuvant chemo(radio)therapy.

The incidence of postoperative complications was 19.3%, and most of the postoperative complications were grade I-II complications. Infection, intestinal obstruction and anastomotic stenosis were the main postoperative complications. All the patients with postoperative complications were discharged smoothly with conservative symptomatic treatment. Lupattelli et al. published a multicenter retrospective study in which 76 patients with locally advanced rectal cancer were given 45 Gy (25 times) in the pelvis, and the local tumor dose was increased to 52.5 to 57.5 Gy (25 times), and the recent results showed that the pCR rate was 27.8%, the incidence of grade 3-4 adverse reactions was 10.5%, and the incidence of surgical complications was 18.1% (42). Based on the above results, we speculated that neoadjuvant immunotherapy with or without neoadjuvant chemo(radio)therapy did not significantly increase postoperative complications, but many studies were still needed to confirm.

### Limitations

The meta-analysis had several limitations. Firstly, neoadjuvant immunotherapy regimens were inconsistent among the included articles, which could affect the results. Whether it should be combined with neoadjuvant chemo(radio)therapy and neoadjuvant chemo(radio)therapy regimens were inconsistent, which was also one of the limitations. Secondly, few related articles on neoadjuvant immunotherapy for colorectal cancer (most of which are single-arm studies with no control group) could be one of the limitations. Thirdly, limited patients and clinical data of neoadjuvant immunotherapy for non-metastatic colorectal cancer could affect the results.

## Conclusion

By using the collected clinical data, we speculated that neoadjuvant immunotherapy could increase MPR and pCR rate, especially for dMMR/MSI-H status. Neoadjuvant immunotherapy combined with chemo(radio)therapy could enhance the therapeutic effect (MPR and pCR rate). Compared with previous clinical data of neoadjuvant chemo(radio)therapy, neoadjuvant immunotherapy did not increase the incidence of postoperative complications and adverse events. We expected the precise neoadjuvant immunotherapy regimens with or without chemo(radio)therapy would appear, which could reduce postoperative complications and adverse events, increase MPR, pCR rate and other outcomes. At the same time, we also looked forward to the emergence of more RCTs that can confirm the clinical effects of neoadjuvant immunotherapy for non-metastatic colorectal cancer. Neoadjuvant immunotherapy could be another treatment option for non-metastatic colorectal cancer treatment.

## Data availability statement

The original contributions presented in the study are included in the article/**Supplementary Material**. Further inquiries can be directed to the corresponding author.

## Author contributions

All authors participated in the study. LZ, G-YZ and F-JW performed the literature search and the acquisition of data. LZ and F-JW performed data analysis. X-QY and XL participated in the interpretation of data and paper writing. LZ and XL were responsible for the editing, revision and submission of the paper. All authors contributed to the article and approved the submitted version.
