# Supplementary material for: Meta-analysis of neoadjuvant immunotherapy for non-metastatic colorectal cancer
Source: Front Immunol. 2023 Jan 27;14:1044353. doi: 10.3389/fimmu.2023.1044353 (PMC9911889; doi:10.3389/fimmu.2023.1044353)
Supplement: Supplementary file 1 [file DataSheet_1.zip › Supplementary Material 1.pdf]

National Institute for Health Research

UNIVERSITY of York
Centre for Reviews and Dissemination

Systematic review

A list of fields that can be edited in an update can be found here

1. * Review title.

Give the title of the review in English

Meta-analysis of neoadjuvant immunotherapy for non-metastatic colorectal cancer

2. Original language title.

For reviews in languages other than English, give the title in the original language. This will be displayed with the English language title.

Meta-analysis of neoadjuvant immunotherapy for non-metastatic colorectal cancer

3. * Anticipated or actual start date.

Give the date the systematic review started or is expected to start.

06/08/2022

4. * Anticipated completion date.

Give the date by which the review is expected to be completed.

30/11/2022

5. * Stage of review at time of this submission.

This field uses answers to initial screening questions. It cannot be edited until after registration.

Tick the boxes to show which review tasks have been started and which have been completed.

Update this field each time any amendments are made to a published record.

The review has not yet started: Yes

Review stage	Started	Completed
Preliminary searches	No	No
Piloting of the study selection process	No	No
Formal screening of search results against eligibility criteria	No	No
Data extraction	No	No
Risk of bias (quality) assessment	No	No
Data analysis	No	No

Provide any other relevant information about the stage of the review here.

6. * Named contact.

The named contact is the guarantor for the accuracy of the information in the register record. This may be any member of the review team.

Xin Liu

Email salutation (e.g. "Dr Smith" or "Joanne") for correspondence:

Mr Liu

7. * Named contact email.

Give the electronic email address of the named contact.

liuxin5626855@sina.com

8. Named contact address

Give the full institutional/organisational postal address for the named contact.

Cancer hospital of China medical university, Liaoning cancer hospital and institute\nshenyang, xiao he yan road, No.44, Liaoning Province CHINA

9. Named contact phone number.

Give the telephone number for the named contact, including international dialling code.

8618900918981

10. * Organisational affiliation of the review.

Full title of the organisational affiliations for this review and website address if available. This field may be completed as 'None' if the review is not affiliated to any organisation.

Cancer Hospital of China Medical University. Liaoning Cancer Hospital and Institute

Organisation web address:

11. * Review team members and their organisational affiliations.

Give the personal details and the organisational affiliations of each member of the review team. Affiliation refers to groups or organisations to which review team members belong. **NOTE: email and country now MUST be entered for each person, unless you are amending a published record.**

Mr Xin Liu. Cancer Hospital of China Medical University. Liaoning Cancer Hospital and Institute
Mr Long Zhou. Department of Orthopedics, Shengjing Hospital of China Medical University
Mr Xiao-Quan Yang. Department of general surgery, Cancer Hospital of China Medical University
Mr Feng-jian Wang. Department of Colorectal surgery, Cancer Hospital of China Medical University
Mr Guang-yue Zhao. Department of Colorectal surgery, Cancer Hospital of China Medical University

12. * Funding sources/sponsors.

Details of the individuals, organizations, groups, companies or other legal entities who have funded or sponsored the review.

No

Grant number(s)

State the funder, grant or award number and the date of award

No

13. * Conflicts of interest.

List actual or perceived conflicts of interest (financial or academic).

None

14. Collaborators.

Give the name and affiliation of any individuals or organisations who are working on the review but who are not listed as review team members. **NOTE: email and country must be completed for each person, unless you are amending a published record.**

15. * Review question.

State the review question(s) clearly and precisely. It may be appropriate to break very broad questions down into a series of related more specific questions. Questions may be framed or refined using PI(E)COS or similar where relevant.

The PICO model was followed to guide our literature research in the subgroup analysis: population, intervention, comparator and outcomes. The population included non-metastatic colorectal cancer patients. The intervention was dMMR group. The comparator was pMMR group. The outcomes included pCRs and MPR.

16. * Searches.

State the sources that will be searched (e.g. Medline). Give the search dates, and any restrictions (e.g. language or publication date). Do NOT enter the full search strategy (it may be provided as a link or attachment below.)

According to the design and purpose of the article, we conducted the relevant literature search in the Embase, PubMed, Cochrane Library, CNKI (China National Knowledge Infrastructure) and Wanfang databases (up to August 2022). The search terms were “colorectal cancer” and “neoadjuvant

immunotherapy”. (supplementary material 5).

17. URL to search strategy.

Upload a file with your search strategy, or an example of a search strategy for a specific database, (including the keywords) in pdf or word format. In doing so you are consenting to the file being made publicly accessible. Or provide a URL or link to the strategy. Do NOT provide links to your search **results**.

https://PubMed.ncbi.nlm.nih.gov/?term=colorectal+cancer+and+neoadjuvant+immunotherapy

Alternatively, upload your search strategy to CRD in pdf format. Please note that by doing so you are consenting to the file being made publicly accessible.

Do not make this file publicly available until the review is complete

18. * Condition or domain being studied.

Give a short description of the disease, condition or healthcare domain being studied in your systematic review.

The incidence of colorectal cancer is high, and it create a serious threat to human health [1]. Neoadjuvant therapy has been widely used in the clinical treatment, and it is one of the important modes of colorectal cancer [2-3]. Neoadjuvant therapy for rectal cancer is currently based on neoadjuvant radiotherapy, combined with chemotherapy drugs, while neoadjuvant therapy for colon cancer and resectable metastatic colorectal cancer is mostly based on chemotherapy drugs and targeted drugs [4-5]. ORR (objective response rate) and pCR (pathological complete response) rate of colorectal cancer after neoadjuvant chemotherapy are 40% and 5% respectively, while the pCR rate of colorectal cancer after neoadjuvant chemotherapy chemoradiotherapy is about 10%-15% [6-7].

The original NICHE study reported the final efficacy data at the 2022 ASCO Annual Meeting, it showed that 30% of pMMR or MSS and 100% of dMMR or MSI-H colorectal cancer could respond to neoadjuvant nivolumab plus ipilimumab. However, the clinical efficacy and scope of neoadjuvant immunotherapy for colorectal cancer remains unclear. Therefore, we collected relevant articles of neoadjuvant immunotherapy for colorectal cancer. We tried to explain the clinical effects of neoadjuvant immunotherapy for colorectal cancer and further compared the difference between dMMR group and pMMR group.

19. * Participants/population.

Specify the participants or populations being studied in the review. The preferred format includes details of both inclusion and exclusion criteria.

non-metastatic colorectal cancer patients

20. * Intervention(s), exposure(s).

Give full and clear descriptions or definitions of the interventions or the exposures to be reviewed. The preferred format includes details of both inclusion and exclusion criteria.

dMMR group

21. * Comparator(s)/control.

Where relevant, give details of the alternatives against which the intervention/exposure will be compared (e.g. another intervention or a non-exposed control group). The preferred format includes details of both inclusion and exclusion criteria.

pMMR group

22. * Types of study to be included.

Give details of the study designs (e.g. RCT) that are eligible for inclusion in the review. The preferred format includes both inclusion and exclusion criteria. If there are no restrictions on the types of study, this should be stated.

single-arm study, cohort study, prospective study, retrospective study and RCTs

23. Context.

Give summary details of the setting or other relevant characteristics, which help define the inclusion or exclusion criteria.

The inclusion criteria were as follows: (1) non-metastatic colorectal cancer, (2) single-arm study, cohort study, prospective study, retrospective study and RCTs, (3) the included patients performed neoadjuvant immunotherapy.

The exclusion criteria were as follows: (1) metastatic colorectal cancer; (2) case reports, meeting, letter and other unsuitable types; (3) no neoadjuvant immunotherapy.

24. * Main outcome(s).

Give the pre-specified main (most important) outcomes of the review, including details of how the outcome is defined and measured and when these measurement are made, if these are part of the review inclusion criteria.

pCRs, MPR, ORR, R0-resection and Anus preserving rate

Measures of effect

Please specify the effect measure(s) for you main outcome(s) e.g. relative risks, odds ratios, risk difference, and/or 'number needed to treat.

Stata 11.0 and RevMan 5.0 software was used to analyze the dichotomous data, and it was evaluated by relative risks (ORs or RRs) with 95% confidence intervals. Random effects models and fixed effects model were used to analyse the data with huge heterogeneity (I^2?50%) and for little heterogeneity ($I^2$50%) respectively. Publication bias was assessed by the funnel plots.

25. * Additional outcome(s).

List the pre-specified additional outcomes of the review, with a similar level of detail to that required for main outcomes. Where there are no additional outcomes please state 'None' or 'Not applicable' as appropriate to the review

No

Measures of effect

Please specify the effect measure(s) for you additional outcome(s) e.g. relative risks, odds ratios, risk difference, and/or 'number needed to treat.

26. * Data extraction (selection and coding).

Describe how studies will be selected for inclusion. State what data will be extracted or obtained. State how this will be done and recorded.

Two reviewers (GYZ and FJW) searched the relevant literatures and sorted the useful clinical data independently with the help of the revised version of MINORS (methodological index for non-randomized studies) and NOS scores guidelines [17-18]. The third reviewer (LZ) resolved the inconsistencies between the above two authors.

27. * Risk of bias (quality) assessment.

State which characteristics of the studies will be assessed and/or any formal risk of bias/quality assessment tools that will be used.

The revised version of MINORS was used for the quality assessment of observational or non-randomized studies, while NOS scores guideline was used for the quality assessment of controlled clinical or cohort studies. The third reviewer (LZ) resolved the inconsistencies between the above two authors.

28. * Strategy for data synthesis.

Describe the methods you plan to use to synthesise data. This **must not be generic text** but should be **specific to your review** and describe how the proposed approach will be applied to your data. If meta-analysis is planned, describe the models to be used, methods to explore statistical heterogeneity, and software package to be used.

Stata 11.0 and RevMan 5.0 software was used to analyze the dichotomous data, and it was evaluated by relative risks (ORs or RRs) with 95% confidence intervals. Random effects models and fixed effects model were used to analyse the data with huge heterogeneity (I^2?50%) and for little heterogeneity ($I^2$50%) respectively. Publication bias was assessed by the funnel plots.

29. * Analysis of subgroups or subsets.

State any planned investigation of ‘subgroups’. Be clear and specific about which type of study or participant will be included in each group or covariate investigated. State the planned analytic approach.

The PICO model was followed to guide our literature research in the subgroup analysis: population, intervention, comparator and outcomes. The population included non-metastatic colorectal cancer patients. The intervention was dMMR group. The comparator was pMMR group. The outcomes included pCRs and MPR.

30. * Type and method of review.

Select the type of review, review method and health area from the lists below.

Type of review

Cost effectiveness
No

Diagnostic
No

Epidemiologic
No

Individual patient data (IPD) meta-analysis
No

Intervention
Yes

Living systematic review
No

Meta-analysis
No

Methodology
No

Narrative synthesis
No

Network meta-analysis
No

Pre-clinical
No

Prevention
No

Prognostic
No

Prospective meta-analysis (PMA)
No

Review of reviews
No

Service delivery
No

Synthesis of qualitative studies
No

Systematic review
Yes

Other
No

Health area of the review

Alcohol/substance misuse/abuse
No

Blood and immune system
No

Cancer
Yes

Cardiovascular

No

Care of the elderly
No

Child health
No

Complementary therapies
No

COVID-19
No

Crime and justice
No

Dental
No

Digestive system
No

Ear, nose and throat
No

Education
No

Endocrine and metabolic disorders
No

Eye disorders
No

General interest
No

Genetics
No

Health inequalities/health equity
No

Infections and infestations
No

International development
No

Mental health and behavioural conditions
No

Musculoskeletal
No

Neurological
No

Nursing
No

Obstetrics and gynaecology
No

Oral health
No

Palliative care
No

Perioperative care
No

Physiotherapy
No

Pregnancy and childbirth
No

Public health (including social determinants of health)
No

Rehabilitation
No

Respiratory disorders
No

Service delivery
No

Skin disorders
No

Social care
No

Surgery
No

Tropical Medicine
No

Urological
No

Wounds, injuries and accidents
No

Violence and abuse
No

31. Language.

Select each language individually to add it to the list below, use the bin icon to remove any added in error.
English

There is not an English language summary

32. * Country.

Select the country in which the review is being carried out. For multi-national collaborations select all the countries involved.

China

33. Other registration details.

Name any other organisation where the systematic review title or protocol is registered (e.g. Campbell, or The Joanna Briggs Institute) together with any unique identification number assigned by them. If extracted data will be stored and made available through a repository such as the Systematic Review Data Repository (SRDR), details and a link should be included here. If none, leave blank.

No

34. Reference and/or URL for published protocol.

If the protocol for this review is published provide details (authors, title and journal details, preferably in Vancouver format)

No

Add web link to the published protocol.

Or, upload your published protocol here in pdf format. Note that the upload will be publicly accessible.

No I do not make this file publicly available until the review is complete

Please note that the information required in the PROSPERO registration form must be completed in full even if access to a protocol is given.

35. Dissemination plans.

Do you intend to publish the review on completion?

No

Give brief details of plans for communicating review findings.?

36. Keywords.

Give words or phrases that best describe the review. Separate keywords with a semicolon or new line. Keywords help PROSPERO users find your review (keywords do not appear in the public record but are included in searches). Be as specific and precise as possible. Avoid acronyms and abbreviations unless these are in wide use.

neoadjuvant immunotherapy, non-metastatic colorectal cancer, meta-analysis

37. Details of any existing review of the same topic by the same authors.

If you are registering an update of an existing review give details of the earlier versions and include a full bibliographic reference, if available.

No

38. * Current review status.

Update review status when the review is completed and when it is published.New registrations must be ongoing so this field is not editable for initial submission.
Please provide anticipated publication date

Review_Ongoing

39. Any additional information.

Provide any other information relevant to the registration of this review.

No

40. Details of final report/publication(s) or preprints if available.

Leave empty until publication details are available OR you have a link to a preprint (NOTE: this field is not editable for initial submission). List authors, title and journal details preferably in Vancouver format.

Give the link to the published review or preprint.
