# Supplementary material for: Meta-analysis of neoadjuvant immunotherapy for non-metastatic colorectal cancer
Source: Front Immunol. 2023 Jan 27;14:1044353. doi: 10.3389/fimmu.2023.1044353 (PMC9911889; doi:10.3389/fimmu.2023.1044353)
Supplement: Supplementary file 1 [file DataSheet_1.zip › Supplementary Material 3.doc]

**Supplementary material 3:** clinical stage, T stage, N stage and M stage of the included articles

| Study | Clinical stage | | | | Clinical T stage | | Clinical N stage | | | Clinical M stage | |
| --- | --- | --- | --- | --- | --- | --- | --- | --- | --- | --- | --- |
|  | Ⅰ | Ⅱ | Ⅲ | Ⅳ | T1- T2 | T3- T4 | N0 | N1 | N2 | M0 | M1 |
| Bando H 2022 | 0 | 33 | 11 | 0 | 0 | 44 | NR | NR | NR | NR | NR |
| Chalabi M 2022 | 7 | 9 | 24 | 0 | NR | NR | NR | NR | NR | 0 | 0 |
| Hu H 2022 | 0 | 4 | 32 | 0 | 0 | 36 | 4 | 7 | 15 | 0 | 0 |
| Kothari A 2022 | 0 | 1 | 8 | 0 | 0 | 9 | 1 | 3 | 5 | 0 | 0 |
| Li YJ 2021 | 0 | 0 | 24 | 0 | 0 | 24 | 0 | 9 | 15 | 0 | 0 |
| Lin Z 2021 | 0 | 4 | 26 | 0 | 0 | 30 | 4 | 16 | 10 | 0 | 0 |
| Liu ZX 2022 | 0 | 12 | 82 | 0 | 0 | 94 | 12 | NR | NR | 0 | 0 |
| Rahma OE 2021 | NR | NR | NR | NR | 4 | 86 | 20 | 35 | 35 | 0 | 0 |
| Shamseddine A 2020 | 0 | 1 | 12 | 0 | NR | 12 | 0 | 5 | 7 | 0 | 0 |
| Zhang X 2022 | 0 | 4 | 28 | 0 | 0 | 32 | 4 | 5 | 23 | 0 | 0 |

**Notes: dMMR: deficient mismatch repair; MSI-H: microsatellite instability-high; MSI-L: microsatellite instability-low; pMMR: proficient mismatch repair; MSS: microsatellite stable; CRM: circumferential resection margin; EMVI: extramural venous invasion; pCR: pathological complete response; MPR: major pathological response; ORR: objective response rate; cCR: complete clinical response; NR: no record.**

**The orders of additional information were range, standard deviation, percentage or NR (if not reported).**
