# Supplementary material for: Meta-analysis of neoadjuvant immunotherapy for non-metastatic colorectal cancer
Source: Front Immunol. 2023 Jan 27;14:1044353. doi: 10.3389/fimmu.2023.1044353 (PMC9911889; doi:10.3389/fimmu.2023.1044353)
Supplement: Supplementary file 1 [file DataSheet_1.zip › Supplementary Material 4.doc]

**Supplementary material 4: pathological stage of the included articles**

| Study | Pathological stage | | | | Clinical T stage | | | | | | Clinical N stage | | |
| --- | --- | --- | --- | --- | --- | --- | --- | --- | --- | --- | --- | --- | --- |
|  | 0 | Ⅰ | Ⅱ | Ⅲ | Tis | T0 | T1 | T2 | T3 | T4 | N0 | N1 | N2 |
| Bando H 2022 | NR | NR | NR | NR | NR | NR | NR | NR | NR | NR | NR | NR | NR |
| Chalabi M 2022 | NR | NR | NR | NR | NR | NR | NR | NR | NR | NR | NR | NR | NR |
| Hu H 2022 | 30  (83.3) | 2  (5.5) | 0 | 4  (11.1) | 2  (5.5) | 29  (80.5) | 1  (2.7) | 1  (2.7) | 3  (8.3) | 0 | 35  (97.2) | 1  (2.7) | 0 |
| Kothari A 2022 | NR | NR | NR | NR | NR | NR | NR | NR | NR | NR | NR | NR | NR |
| Li YJ 2021 | NR | NR | NR | NR | NR | NR | NR | NR | NR | NR | NR | NR | NR |
| Lin Z 2021 | 14  (51.9) | 0 | 5  (18.5) | 8  (29.6) | 1  (3.7) | 13  (48.1) | NR | 5  (18.5) | 8  (29.6) | NR | 19  (70.4) | 6  (22.2) | 2  (7.4) |
| Liu ZX 2022 | NR | NR | NR | NR | NR | NR | NR | NR | NR | NR | NR | NR | NR |
| Rahma OE 2021 | NR | NR | NR | NR | NR | NR | NR | NR | NR | NR | NR | NR | NR |
| Shamseddine A 2020 | 3  (25) | 1  (8.3) | 4  (33.3) | 4  (33.3) | 0 | 3  (25) | 0 | 1  (8.3) | 7  (58.3) | 1  (8.3) | 8  (66.6) | 2  (16.7) | 2  (16.7) |
| Zhang X 2022 | 22  (75.9) | 6  (20.6) | 1  (3.5) | 0 | 0 | 22  (75.9) | 3  (10.3) | 3  (10.3) | 1  (3.5) | 0 | 29  (100) | 0 | 0 |

**Notes: dMMR: deficient mismatch repair; MSI-H: microsatellite instability-high; MSI-L: microsatellite instability-low; pMMR: proficient mismatch repair; MSS: microsatellite stable; CRM: circumferential resection margin; EMVI: extramural venous invasion; pCR: pathological complete response; MPR: major pathological response; ORR: objective response rate; cCR: complete clinical response; NR: no record.**

**The orders of additional information were range, standard deviation, percentage or NR (if not reported).**
