# Supplementary material for: Meta-analysis of neoadjuvant immunotherapy for non-metastatic colorectal cancer
Source: Front Immunol. 2023 Jan 27;14:1044353. doi: 10.3389/fimmu.2023.1044353 (PMC9911889; doi:10.3389/fimmu.2023.1044353)
Supplement: Supplementary file 1 [file DataSheet_1.zip › Supplementary Material 5.doc]

**Supplementary material 5:** postoperative complications of the included studies

| Study | infection | bleeding | anastomotic stenosis | enteritis | ileus | Total | | |
| --- | --- | --- | --- | --- | --- | --- | --- | --- |
|  |  |  |  |  |  | Sum | Ⅰ-Ⅱ | Ⅲ-Ⅳ |
| Bando H 2022 | 4 | NR | NR | NR | 4 | 12(31.5) | NR | NR |
| Chalabi M 2022 | NR | NR | NR | NR | NR | NR | NR | NR |
| Hu H 2022 | NR | 2 | 1 | 1 | NR | 5(13.8) | 5(13.8) | 0 |
| Kothari A 2022 | NR | NR | NR | NR | NR | NR | NR | NR |
| Li YJ 2021 | 1 | 0 | 3 | 1 | NR | 5(25) | NR | NR |
| Lin Z 2021 | 3 | 1 | 0 | 0 | NR | 4(14.8) | NR | NR |
| Liu ZX 2022 | NR | NR | NR | NR | NR | 21(22.3) | 13(13.8) | 8(8.5) |
| Rahma OE 2021 | NR | NR | NR | NR | NR | NR | NR | NR |
| Shamseddine A 2020 | NR | NR | NR | NR | NR | NR | NR | NR |
| Zhang X 2022 | 1 | 2 | NR | NR | NR | 3(10.3) | 3(10.3) | 0 |

**Notes: dMMR: deficient mismatch repair; MSI-H: microsatellite instability-high; MSI-L: microsatellite instability-low; pMMR: proficient mismatch repair; MSS: microsatellite stable; CRM: circumferential resection margin; EMVI: extramural venous invasion; pCR: pathological complete response; MPR: major pathological response; ORR: objective response rate; cCR: complete clinical response; NR: no record.**

**The orders of additional information were range, standard deviation, percentage or NR (if not reported).**
