# Supplementary material for: Meta-analysis of neoadjuvant immunotherapy for non-metastatic colorectal cancer
Source: Front Immunol. 2023 Jan 27;14:1044353. doi: 10.3389/fimmu.2023.1044353 (PMC9911889; doi:10.3389/fimmu.2023.1044353)
Supplement: Supplementary file 1 [file DataSheet_1.zip › Supplementary Material 6.doc]

**Supplementary material 6: the details of adverse events to neoadjuvant immunotherapy and chemoradiotherapy**

**adverse events of chemoradiotherapy**

| Study | Leukopenia | | Elevated transaminases | | Gastrointertinal disorders | | Total | |
| --- | --- | --- | --- | --- | --- | --- | --- | --- |
|  | Ⅰ-Ⅱ | Ⅲ-Ⅳ | Ⅰ-Ⅱ | Ⅲ-Ⅳ | Ⅰ-Ⅱ | Ⅲ-Ⅳ | Ⅰ-Ⅱ | Ⅲ-Ⅳ |
| Bando H 2022 | NR | NR | NR | NR | NR | NR | NR | NR |
| Chalabi M 2022 | NR | NR | NR | NR | NR | NR | NR | NR |
| Hu H 2022 | NR | NR | NR | NR | NR | NR | NR | NR |
| Kothari A 2022 | NR | NR | NR | NR | NR | NR | NR | NR |
| Li YJ 2021 | NR | NR | NR | NR | NR | NR | 6(25) | 0 |
| Lin Z 2021 | 24 | 1 | 18 | 0 | 15 | 0 | 29(96.7) | 8(26.7) |
| Liu ZX 2022 | 18 | 3 | 15 | 6 | 16 | 2 | 21(22.3) | NR |
| Rahma OE 2021 | NR | NR | NR | NR | NR | NR | 39(48.2) | 0 |
| Shamseddine A 2020 | 1 | 0 | NR | NR | 9 | NR | NR | NR |
| Zhang X 2022 | NR | NR | NR | NR | NR | NR | NR | NR |

**immune-related adverse events**

| Study | Hypothyroidism | | Dermatitis | | Reactive cutaneous capillary endothelial proliferation | | Gastrointertinal disorders | | Total | |
| --- | --- | --- | --- | --- | --- | --- | --- | --- | --- | --- |
|  | Ⅰ-Ⅱ | Ⅲ-Ⅳ | Ⅰ-Ⅱ | Ⅲ-Ⅳ | Ⅰ-Ⅱ | Ⅲ-Ⅳ | Ⅰ-Ⅱ | Ⅲ-Ⅳ | Ⅰ-Ⅱ | Ⅲ-Ⅳ |
| Bando H 2022 | NR | NR | NR | NR | NR | NR | NR | NR | NR | NR |
| Chalabi M 2022 | NR | NR | NR | NR | NR | NR | NR | NR | NR | NR |
| Hu H 2022 | 4 | 0 | 4 | 0 | 5 | 0 | 4 | 0 | 26(76) | 0 |
| Kothari A 2022 | NR | NR | NR | NR | NR | NR | NR | NR | NR | NR |
| Li YJ 2021 | 2 | 0 | 1 | 0 | 0 | 0 | 0 | 0 | 3(12.5) | 0 |
| Lin Z 2021 | 2 | 0 | 0 | 0 | 22 | 0 | 0 | 0 | 22(81.5) | 0 |
| Liu ZX 2022 | 20 | 1 | 35 | 0 | NR | NR | 10 | 0 | 35(37.2) | 1(1.06) |
| Rahma OE 2021 | 7 | 1 | 3 | 0 | NR | NR | 19 | 2 | 35(43.2) | 3(3.7) |
| Shamseddine A 2020 | NR | NR | NR | NR | NR | NR | NR | NR | NR | NR |
| Zhang X 2022 | 1 | 0 | 1 | 0 | NR | NR | 1 | 0 | 10(31.2) | 0 |

**Notes: dMMR: deficient mismatch repair; MSI-H: microsatellite instability-high; MSI-L: microsatellite instability-low; pMMR: proficient mismatch repair; MSS: microsatellite stable; CRM: circumferential resection margin; EMVI: extramural venous invasion; pCR: pathological complete response; MPR: major pathological response; ORR: objective response rate; cCR: complete clinical response; NR: no record.**

**The orders of additional information were range, standard deviation, percentage or NR (if not reported).**
