# Supplementary material for: Meta-analysis of neoadjuvant immunotherapy for non-metastatic colorectal cancer
Source: Front Immunol. 2023 Jan 27;14:1044353. doi: 10.3389/fimmu.2023.1044353 (PMC9911889; doi:10.3389/fimmu.2023.1044353)
Supplement: Supplementary file 1 [file DataSheet_1.zip › Supplementary Material 7.doc]

**Supplementary material 7: the details of neoadjuvant immunotherapy plan**

| Study | **Neoadjuvant immunotherapy** |
| --- | --- |
| Bando H 2022 | The study treatments included five cycles of nivolumab monotherapy (240 mg every 2 weeks) and subsequent radical surgery using a sphincter-sparing procedure or abdominoperineal resection with TME, after CRT with capecitabine and radiation to a dose of 50.4 Gy, in 28 fractions. We planned to start nivolumab within 14 days of completion of preoperative CRT; the period between the end of CRT and radical surgery was planned for 12 weeks (Supplementary Fig. S1). For patients with favorable postoperative conditions, a maximum of 6 months on adjuvant treatment with mFOLFOX6 or CAPOX was recommended, at the investigator’s discretion. |
| Chalabi M 2022 | ipilimumab 1mg/kg on D1 + nivolumab 3mg/kg on D1 and D15 (dMMR); ipilimumab 1mg/kg on D1 + nivolumab 3mg/kg on D1 and D15 + celecoxib 200mg daily from D1 until the day before surgery (pMMR); The median duration from the first dose of nivolumab to surgery was 32days. |
| Hu H 2022 | In both treatment groups, toripalimab 3 mg/kg (Junshi BioSciences, Shanghai, China) was given intravenously over 30 min on day 1 of each 14-day cycle, for six cycles, before surgical resection. Patients in the toripalimab plus celecoxib group were also given celecoxib 200 mg (Pfizer, New York, NY, USA) orally twice daily from day 1 to 14 each 14-day cycle. Surgery was planned for within 4 weeks after the last neoadjuvant toripalimab dose. Dose modifications were not permitted, but toripalimab or celecoxib, or both, could be interrupted or delayed for a maximum of 2 weeks. |
| Kothari A 2022 | Four patients received chemotherapy before initiation of CPI treatment, with a mean of four cycles of treatment. The median number of immunotherapy treatment doses was 5 (range 1–16).  The median interval from initiation of CPI therapy to surgery was 5 months, and the interval between the last dose of CPI and surgery was 48 days. |
| Li YJ 2021 | Immunotherapy (PD-1 antibody 200 mg)+induction chemotherapy(CapeOx regimen: oxaliplatin 130 mg/m^2^, intravenous infusion, on the first day, capecitabine 1 000 mg/m^2^, orally, twice a day, on days 1 to 14); once every 3 weeks, for a total of 3 cycles)+ IMRT (Gross tumor volume-GTV: 50.6 Gy/clinical target volume-CTV 41.8 Gy/22 f) . Surgery is performed 4-6 weeks after neoadjuvant therapy. |
| Lin Z 2021 | Eligible patients received SCRT (5×5 Gy over 5 days), followed 1 week later by two subsequent 21-day cycles of CAPOX (oxaliplatin 130 mg/m^2^ intravenously, day 1; capecitabine 1000 mg/m^2^ oral twice daily, days 1–14) plus camrelizumab (200 mg intravenous drip, day 1), followed by radical surgery after 1 week. |
| Liu ZX 2022 | pMMR and MSS were treated with CapeOx (2 cycles) combined with PD-1 single agent (2 cycles), long-course radiotherapy±CapeOx (2 cycles), dMMR or MSI-H patients were treated with PD-1 single agent (2 cycles)±long-course radiotherapy for rectal cancer; PD-1 single agent for colon cancer. |
| Rahma OE 2021 | Patients were randomized to receive 6 cycles of FOLFOX (5-fluorouracil, leucovorin, and oxaliplatin) followed by chemoradiotherapy (825 mg/m^2^ of capecitabine twice daily concurrently with 4500 cGy in 25 fractions for 5 weeks plus a 540-cGy boost in 3 fractions) starting 3 to 4 weeks after FOLFOX (CA) or the same dosage of FOLFOX followed by the same chemoradiotherapy regimen in combination with 200mg of pembrolizumab every 3 weeks starting on day 1 of chemoradiotherapy for up to 6 doses (PA). Surgery was performed 8 to 12 weeks after the last dose of radiotherapy. |
| Shamseddine A 2020 | During the first week, patients underwent short course radiation therapy. Prior to radiotherapy patients underwent 3 dimensional CT simulation. Intensity modulated for treatment delivery. Treatment volumes were defined as follows: GTV includes all primary tumor extent, and enlarged lymph nodes, CTV includes GTV with 0.5 cm extension and all perirectal, presacral, and internal iliac lymph nodes all the way up to the sacral promontory, PTV is a 1 cm expansion of the CTV in all directions. Prescription dose was 25 Gy (prescribed to PTV) delivered over 5 fractions. After one week, patients received the first cycle of mFOLFOX6 (oxaliplatin 85 mg/m^2^ in a 2-h infusion, leucovorin 400 mg/m^2^ over 2 h and 48-h infusion of fluorouracil 2400 mg/m^2^) 30  min after avelumab (10 mg/Kg). The same regimen was repeated every 2 weeks for 6 cycles. Then 3-4 weeks after the last cycle, patients underwent total mesorectal excision. |
| Zhang X 2022 | All 32 patients who met the inclusion and exclusion criteria received nIT with single-agent PD-1 inhibitor, of which 4 patients used pembrolizumab, 9 patients used sintilimab and 19 patients treated with tiselizumab. Within the first day of each treatment cycle, they received nIT with intravenous drip of 200mg PD-1 inhibitor. The duration of each cycle of treatment was 3 weeks, regardless of how many courses of treatment were used (200 mg IV Q3W). Before using nIT, regardless of whether you have received nCT or nCRT. All patients underwent radical resection or W&W strategy. Of the 29 patients who underwent surgery, 22 received adjuvant immunotherapy with PD-1 blockade. |

**Notes: dMMR: deficient mismatch repair; MSI-H: microsatellite instability-high; MSI-L: microsatellite instability-low; pMMR: proficient mismatch repair; MSS: microsatellite stable; CRM: circumferential resection margin; EMVI: extramural venous invasion; pCR: pathological complete response; MPR: major pathological response; ORR: objective response rate; cCR: complete clinical response; SBRT: stereotactic body radiation therapy; IMRT: implementation of intensity-modulated radiotherapy; NR: no record.**

**The orders of additional information were range, standard deviation, percentage or NR (if not reported).**
