# Supplementary material for: Meta-analysis of neoadjuvant immunotherapy for non-metastatic colorectal cancer
Source: Front Immunol. 2023 Jan 27;14:1044353. doi: 10.3389/fimmu.2023.1044353 (PMC9911889; doi:10.3389/fimmu.2023.1044353)
Supplement: Supplementary file 1 [file DataSheet_1.zip › Supplementary Material 8.doc]

**Supplementary material 8: MINORS score of the included articles**

| Criterion | Study | | | | | | | | | |
| --- | --- | --- | --- | --- | --- | --- | --- | --- | --- | --- |
|  | Bando H 2022 | Chalabi M 2022 | Hu H 2022 | Kothari A  2022 | Li YJ  2021 | Lin Z  2021 | Liu ZX  2022 | Rahma OE 2021 | Shamseddine A  2020 | Zhang X  2022 |
| 1. A stated aim of the study | 2 | 2 | 2 | 2 | 2 | 2 | 2 | 2 | 2 | 2 |
| 2. Inclusion of consecutive patients | 2 | 2 | 2 | 2 | 2 | 2 | 2 | 2 | 2 | 2 |
| 3. Prospective collection of data | 0 | 0 | 0 | 0 | 0 | 0 | 0 | 0 | 0 | 0 |
| 4. Endpoint appropriate to the study aim | 2 | 2 | 2 | 2 | 2 | 2 | 2 | 2 | 2 | 2 |
| 5. Unbiased evaluation of endpoints | 2 | 2 | 2 | 2 | 2 | 2 | 2 | 2 | 2 | 2 |
| 6. Follow-up period appropriate to the major endpoint | 2 | 2 | 2 | 2 | 2 | 2 | 2 | 2 | 2 | 2 |
| 7. Loss to follow up not exceeding 5% | 2 | 2 | 2 | 2 | 2 | 2 | 2 | 2 | 2 | 2 |
| 8. Prospective calculation of the study size: | 0 | 0 | 0 | 0 | 0 | 0 | 0 | 0 | 0 | 0 |
| Sum | 12 | 12 | 12 | 12 | 12 | 12 | 12 | 12 | 12 | 12 |
