# Supplementary material for: Meta-analysis of neoadjuvant immunotherapy for non-metastatic colorectal cancer
Source: Front Immunol. 2023 Jan 27;14:1044353. doi: 10.3389/fimmu.2023.1044353 (PMC9911889; doi:10.3389/fimmu.2023.1044353)
Supplement: Supplementary file 1 [file DataSheet_1.zip › Supplementary Material 9.doc]

**Supplementary material 9: immunotherapy alone vs immunotherapy+nC(R)T**

| Study | immunotherapy alone | | | | immunotherapy+nC(R)T | | | |
| --- | --- | --- | --- | --- | --- | --- | --- | --- |
|  | MPR (%) | | pCRs (%) | | MPR (%) | | pCRs (%) | |
|  | dMMR/MSI-H | pMMR/MSS | dMMR/MSI-H | pMMR/MSS | dMMR/MSI-H | pMMR/MSS | dMMR/MSI-H | pMMR/MSS |
| Bando H 2022 | NR | NR | NR | NR | 3(60) | 14(37.8) | 3(60) | 11(29.7) |
| Chalabi M 2022 | 19(95) | 4(27) | 12(60) | 2(13.5) | NR | NR | NR | NR |
| Hu H 2022 | NR | NR | NR | NR | NR | NR | NR | NR |
| Kothari A 2022 | NR | NR | NR | NR | 9(100) | 0 | 8(888.9) | 0 |
| Li YJ 2021 | NR | NR | NR | NR | NR | NR | NR | NR |
| Lin Z 2021 | NR | NR | NR | NR | 1(100) | 17(65.4) | 1(100) | 12(46.2) |
| Liu ZX 2022 | 17(65.4) | NR | 15(57.5) | NR | NR | 40(58.8) | NR | 24(35.3) |
| Rahma OE 2021 | NR | NR | NR | NR | NR | NR | NR | NR |
| Shamseddine A 2020 | NR | NR | NR | NR | NR | 6(50) | NR | 3(25) |
| Zhang X 2022 | 25(86.2) | 0 | 22(75.9) | 0 | NR | NR | NR | NR |
| Total | 61(81.3) | 4(27) | 49(65.3) | 2(13.5) | 13(86.6) | 77(68.1) | 12(80) | 50(34.9) |

**Notes: dMMR: deficient mismatch repair; MSI-H: microsatellite instability-high; MSI-L: microsatellite instability-low; pMMR: proficient mismatch repair; MSS: microsatellite stable; CRM: circumferential resection margin;**

**EMVI: extramural venous invasion; pCR: pathological complete response; MPR: major pathological response;**

**ORR: objective response rate; cCR: complete clinical response; NR: no record.**

**The orders of additional information were range, standard deviation, percentage or NR (if not reported).**
